# Latitudinal and anthropogenic effects on the structuring of networks linking blood‐feeding flies and their vertebrate hosts

**DOI:** 10.1111/mve.12671

**Published:** 2023-06-01

**Authors:** Ben Bellekom, Owen T. Lewis, Talya D. Hackett

**Affiliations:** ^1^ Department of Biology University of Oxford Oxford UK

**Keywords:** bipartite, biting Diptera, blood meal, ecological interactions, habitat modification

## Abstract

Biting flies (Diptera) transmit pathogens that cause many important diseases in humans as well as domestic and wild animals. The networks of feeding interactions linking these insects to their hosts, and how they vary geographically and in response to human land‐use, are currently poorly documented but are relevant to understanding cross‐species disease transmission. We compiled a database of biting Diptera–host interactions from the literature to investigate how key interaction network metrics vary latitudinally and with human land‐use. Interaction evenness and H2' (a measure of the degree of network specificity) did not vary significantly with latitude. Compared to near‐natural habitats, interaction evenness was significantly lower in agricultural habitats, where networks were dominated by relatively few species pairs, but there was no evidence that the presence of humans and their domesticated animals within networks led to systematic shifts in network structure. We discuss the epidemiological relevance of these results and the implications for predicting and mitigating future spill‐over events.

## Graphical abstract

- Anthropogenic habitat modification significantly impacts network properties of biting Diptera–host networks. Agricultural habitats had significantly lower interaction evenness.
- Neither interaction evenness nor H2' network specialisation varied significantly with latitude.
- There was no evidence that the presence of domestic animals and humans led to altered patterns of interaction evenness.

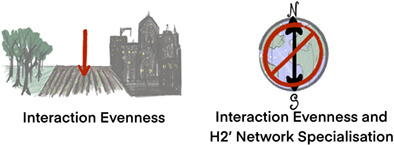

## INTRODUCTION

Across taxa, species richness consistently decreases from equatorial to polar latitudes (Lawrence & Fraser, 2020). This macroecological pattern is linked to changes in the structure and organisation of ecological communities. For example, increased species richness and associated niche partitioning at low latitudes should increase both the frequency and specificity of interspecific interactions such as mutualism, competition and parasitism (Morris et al., 2014; Schemske et al., 2009; Willig et al., 2003). Superimposed on these large‐scale patterns, anthropogenic habitat modification for agriculture and other land‐uses also has marked effects on diversity, community composition and species interactions (Meyer Steiger et al., 2016), and can reshape interactions within a community, even in the absence of biodiversity loss (Morris, 2010; Tylianakis et al., 2007).

Analyses of quantitative ecological networks documenting the interactions among species, weighted by the interaction frequency (Schleuning et al., 2012; Xing & Fayle, 2021), provide an approach for understanding how communities are structured across space and time. A growing number of network studies involving diverse taxa, locations and contexts provide opportunities for synthetic analyses investigating large‐scale ecological patterns (Xing & Fayle, 2021), such as trends in specialisation with latitude (Morris et al., 2014; Schleuning et al., 2012) and in response to land‐use intensification (Tylianakis et al., 2007; Weiner et al., 2014). Few studies, however, have explored the relative influence of large‐scale macroecological gradients and more localised anthropogenic impacts on the structural properties of networks of interacting species (Pellissier et al., 2018; Tylianakis et al., 2007).

Here, we investigate how macro‐ecological and anthropogenic factors influence the properties of networks linking biting flies (Diptera) and their vertebrate hosts. Feeding interactions between Diptera and hosts can now be routinely established using molecular analysis of insect blood meals. These interactions are of particular interest, since biting Diptera are often vectors of a wide range of pathogens including malarial parasites (Sinka et al., 2012), Bluetongue virus (Baker et al., 2021), West Nile virus (Kilpatrick et al., 2005) and Leishmania (Killick‐Kendrick, 1999). These may infect humans, domestic and non‐domestic hosts, and cause significant damage to public and health economics (Barber et al., 2010; Rushton & Lyons, 2015; Sachs & Malaney, 2002). Moreover, their transmission potential can co‐vary with their interactions across land‐use gradients (Meyer Steiger et al., 2016; Müller et al., 2019; Runghen et al., 2021). We analyse biting fly–vertebrate interaction data from a wide range of latitudes and across different habitat types to explore the relative importance of latitude and land‐use in structuring interaction networks.

## METHODS

### Data compilation

Biting Diptera–host interaction data were extracted from the literature on insect blood meals, using a subset of the data compiled by Bellekom et al. (2021). To limit bias, we restricted analyses to data generated using PCR and DNA sequencing (Logue et al., 2016), and excluded studies that used sampling methods and locations inappropriate for collecting a variety of biting Diptera species and subsequent host blood meals. For example, those that used live host‐baited trapping methods, such as livestock and cattle‐baited tents, were excluded as blood meals would be heavily biased towards the bait. Studies that provided data for a single biting Diptera species were also excluded, as these do not provide network data. We also excluded studies lacking site location details. For each remaining study, we recorded site location and classified habitats into three broad categories of anthropogenic landscape modification. Sites where cultivated land or livestock were the dominant land‐use were categorised as Agricultural; those that referenced natural vegetation with limited human presence were classified as Near‐natural; and those where sampling took place within, or around human habitation were classified as Village/Urban. Where sampling was carried out in more than one habitat type, separate networks were generated for each reported habitat. Where habitat could not be determined reliably from the published information (four cases), we used satellite imagery (QGIS in combination with Google Earth) to infer the habitat, using the same categories used for studies where published information on habitat type was available. For example, where satellite imagery indicated that the location of the study was associated with a human settlement, the data were assigned to the Village/Urban category.

Biting Diptera and hosts were resolved to species level, where possible. Where a single species‐level identification was missing for a biting Diptera or host, nodes were simply labelled with the relevant genus or family (e.g., *Culicoides* spp.). Where a genus contained multiple unknown species‐level identifications, we checked whether sympatric congeneric or confamilial species were likely to occur at the focal location using field guides and online resources (GBIF.org, 2021). As before, where no sympatric congeneric or confamilial species occurred, nodes were resolved to the lowest level possible (e.g., *Anas* spp.). Interactions where either the host or the biting insect could not be resolved to genus or family level (or where more than one species could occur in a single node) were removed to prevent different species being combined into the same genus‐level node. In total, 18 biting Diptera and 28 host records were resolved to genus or family level and 76 hosts, 14 biting insects and 119 interactions were removed.

### Data analysis

For each included study, we analysed species interaction data as weighted antagonistic bipartite networks and calculated two network metrics, interaction evenness (IE) and network specialisation (H2’), chosen for their ecological and epidemiological relevance. IE is a weighted network metric based on Shannon diversity that describes the homogeneity of interaction frequencies across all links in the network (E_2_ = H_2_/ln L, where H is Shannon diversity, and L is the number of all links) (Blüthgen et al., 2008; Kaiser‐Bunbury & Blüthgen, 2015). Low IE values indicate contexts in which a small number of species and their links dominate the community (Kaiser‐Bunbury & Blüthgen, 2015). This is particularly relevant if the dominant species are vectors or susceptible hosts. H2' is a weighted network metric that quantifies the deviation of observed interaction frequencies from those expected if interaction frequencies were random (Blüthgen et al., 2006). A high degree of generalism within our network would be expected to facilitate transmission between phylogenetically dissimilar hosts (Abella‐Medrano et al., 2018). IE and H2’ values were calculated using the *networklevel* function in the R package *bipartite* (Dormann et al., 2008).

Variation in host and biting Diptera richness across latitudes was explored using a linear model, including the total number of blood meals analysed per network (a proxy for sampling effort) as a covariate. We explored the extent to which variations in IE and H2’ could be explained by habitat type and latitude using a generalised linear model (GLM) with a Gaussian error distribution. Absolute values for latitude (i.e., removing negative signs) were used to combine data from the northern and southern hemisphere. To control for the confounding effects of network size and species richness, we included species richness (S = number of resource species + number of consumer species), and log‐transformed matrix size (sum of all interactions within the matrix) in the GLM (Galiana et al., 2019). Most of our networks (46 out of 47) comprised Diptera from multiple genera but drawn from a single family; each network could therefore be classified by its dominant family. In the case where multiple families were represented in a network, we classified it by the dominant family (the family with the highest number of interactions). Our models tested for the effects of family as well as the two‐way interaction terms between family and latitude and between family and habitat. This allowed us to examine the influence of Diptera family on IE and H2’ values and how these changed across latitude and between habitat types. Residuals were visually inspected to check model assumptions. The statistical significance of habitat type and latitude was assessed by comparing simpler models to more complex models for variation in deviance based on a chi‐square distribution (Mayi et al., 2020). Post‐hoc analysis was conducted to identify intra‐factor significant differences, using Tukey's HSD (honestly significant difference) tests.

We used a null model to evaluate whether domesticated hosts had a measurable effect on network interaction evenness. Within each network, we simulated targeted removal of humans and domestic animals (specifically chickens, dogs, cats, goats, cattle, horses, pigs, and sheep) and compared this to removal of an equal number of randomly selected host species, both domestic and non‐domestic, replicated 100 times. We then calculated the z‐scores and compared the observed network metric to the distribution of the simulated values.

The dataset used is not an exhaustive set of biting Diptera–host interactions. Therefore to assess sampling completeness for each habitat, we drew species interpolation and extrapolation curves for hosts, biting Diptera and interactions as a function of sampling effort (the number of blood meals analysed) using the iNEXT package (q = 0, data type = incidence frequency) (Hsieh et al., 2016).

All data handling and analysis was conducted using R (version 4.01), and the *bipartite* (Dormann et al., 2008), *iNEXT* and *tidyverse* (Wickham et al., 2019) packages. Figures were plotted using *ggplot2, iNEXT* and *bipartite*.

## RESULTS

**FIGURE 1:**
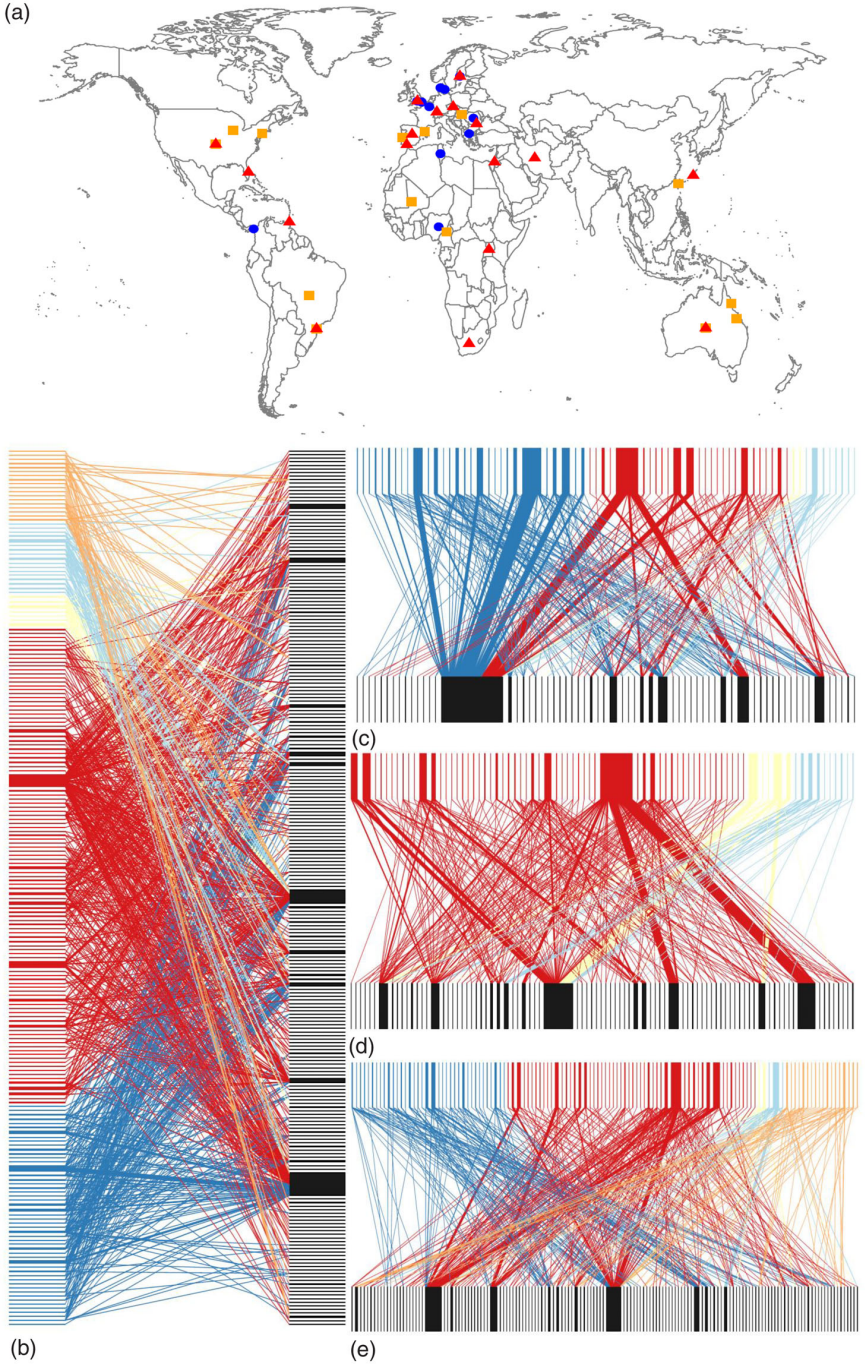
(a) Global distribution of the studies included in the analysis. Agricultural sites are represented by blue circles, Near‐natural sites by red triangles, and Village/Urban sites by yellow squares. Co‐located symbols are studies that sampled in multiple habitat classifications and were treated as separate networks in our analyses (Map data: Google Maps 2020). (b) The aggregated global network containing all host (right) and Diptera (left) interactions, and Diptera (top)–host (bottom) interactions separated by habitat classification: Agricultural(c), Village/Urban (d) and Near‐natural (e). Node and edge widths are proportional to frequency of occurrence and are coloured by biting Diptera family (Ceratopogonidae = dark blue, Culicidae = red, Glossinidae = yellow, Psychodidae = light blue and Simuliidae = orange).

In total, we compiled data for 9102 biting Diptera blood meals from 45 publications involving field sites in 27 countries (Figure 1a). An aggregated global network contained 227 host species, 202 biting Diptera species and 1121 links (Figure 1b). Based on these data, 47 quantitative bipartite networks were constructed from 14 Agricultural (338 links), 18 Near‐natural (461 links) and 15 Village/Urban sites (322 links). Almost all (97%) of our networks comprised Diptera species from a single family: Culicidae (24 cases), Ceratopogonidae (12 cases), Glossinidae (4 cases), Psychodidae (4 cases) and Simuliidae (2 cases). Sampling sites had a wide latitudinal distribution, ranging from Sweden to South Africa (Figure 1a). Total host and Diptera species richness were lower in Agricultural (65 and 64, respectively) than Village/Urban (87 and 74), and Near‐natural (140 and 115) habitats (Figure 1c–e). We found no significant trend in host and biting Diptera richness with latitude after controlling for sampling effort. Accumulation curves showed that host and biting Diptera species were well‐resolved in Agricultural and Village/Urban habitats, with curves approaching an asymptote in each case; host richness in Near‐natural habitats was less complete. Sampling of biting Diptera–host interactions was incomplete for all levels of anthropogenic landscape modification (Figure S1).

### Influence of geographical and anthropogenic factors on biting Diptera–host networks

**FIGURE 2:**
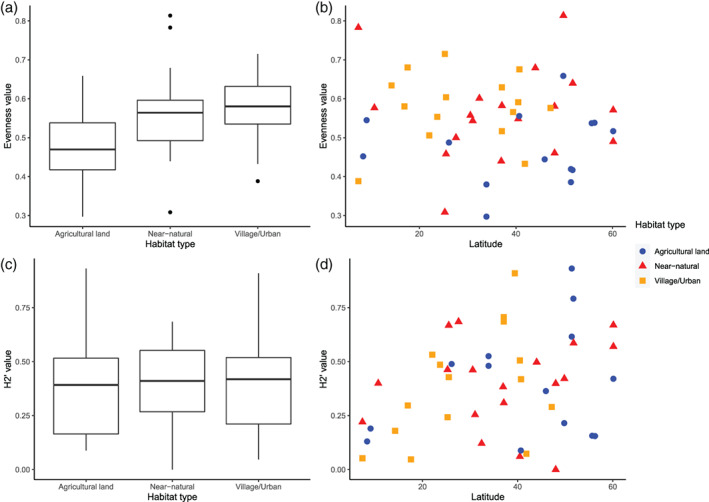
Interaction evenness by habitat type (a) and latitude (b), and H2’ specialisation by habitat type (c) and latitude (d). Interaction evenness was significantly lower in Agricultural habitats. Each box displays the interquartile range and the solid line represents the median. Whiskers display the maximum and minimum interaction evenness and H2’ values for each habitat type.

Interaction evenness differed significantly among habitat types (*X* ^2^ = 0.068, *df* = 2, *p* = 0.042), but did not show a latitudinal trend (*X* ^2^ = 0. 001, *df* = 1, *p* = 0.968) (Figure 2b). Mean interaction evenness was significantly lower in Agricultural habitats (mean = 0.472, SE = 0.026) than in both Village/Urban (mean = 0.576, SE = 0.023, Tukey; *p* = 0.027) and Near‐natural habitats (mean = 0.558, SE = 0.027, Tukey; *p* = 0.040), but did not differ significantly between Village/Urban and Near‐natural habitats (Tukey; *p* = 0.943) (Figure 2a). Species richness (*X* ^2^ = 0.014, *df* = 1, *p* = 0.239), network size (*X* ^2^ = 0.023, *df* = 1, *p* = 0.147) and family (*X* ^2^ = 0.063, *df* = 4, *p* = 0.180) did not explain a significant amount of variance in interaction evenness and there were no significant interactions between family and habitat (*X* ^2^ = 0.045, *df* = 5, *p* = 0.495) or family and latitude (*X* ^2^ = 0.026, *df* = 3, *p* = 0.475).

The average network specialisation (H2’) across our networks was 0.395 (SD = 0.034). Neither latitude (*X* ^2^ = 0.114, *df* = 1, *p* = 0.115) nor habitat (*X* ^2^ = 0.035, *df* = 1, *p* = 0.682) had a significant influence on H2’ (Figure 2c,d). H2’ differed significantly among Diptera families (*X* ^2^ = 0.429, *df* = 4, *p* = 0.030). Simuliidae‐dominated networks had the highest average H2’ (mean = 0.619), followed by Ceratopogonidae (mean = 0.296) and Glossinidae (mean = 0.127). There were no significant interactions between family and habitat (*X* ^2^ = 0.207, *df* = 5, *p* = 0.391) or family and latitude (*X* ^2^ = 0.207, *df* = 3, *p* = 0.242). There was a highly significant decrease in H2’ (*t* = −2.875, *df* = 41, *p* = 0.006) with increasing matrix size.

### The influence of domesticated animals on interaction evenness

Humans and domestic animals were involved in 2928 interactions across Agricultural networks, of which 2653 were Diptera–bovine interactions, involving 87 Diptera species from 5 families (Ceratopogonidae, Glossinidae, Culicidae, Psychodidae and Simuliidae). Near‐natural networks contained 1886 interactions involving humans and domestic animals, and there were 1560 such interactions in Village/Urban networks. There was no evidence that the presence of domesticated animals and humans within networks led to altered patterns of interaction evenness. Resampling networks to target removal of these species did not lead to mean interaction evenness values that differed from those generated when an equivalent number of host species selected at random was removed: Agricultural (mean = 0.721, *n* = 10, SE = 0.472), Village/Urban (mean = 0.696, *n* = 7, SE = 0.434) and Near‐natural (mean = 0.563, *n* = 16, SE = 0.292) (Figure S2).

## DISCUSSION

Overall, latitudinal trends in the structure of biting Diptera–host network properties were dwarfed by the impact of anthropogenic habitat modification. Neither network‐level metric varied significantly with latitude, but agricultural habitats had significantly lower interaction evenness than Near‐natural and Village/Urban habitats.

The absence of a latitudinal trend in feeding specialisation or interaction evenness is counter to the expectation that high species richness at low latitudes will be associated with increased dietary specialisation (Dyer et al., 2007). Turnover in genera of Diptera and hosts was low across latitudes, perhaps explaining the consistency of interspecific interactions. Whilst Diptera families differ in degree of network specialisation as judged by H2’, there was no significant interaction between family and latitude. Consequently, the absence of clear latitudinal trends in network structure, as also documented for host–parasitoid networks (Morris et al., 2014), could result from underlying rules for how these antagonistic interactions are structured, regardless of the diversity and size of component networks, or their taxonomic composition. However, the apparent lack of a latitudinal trend may also result from local climatic differences (e.g., rainfall) among sites at similar latitudes masking latitudinal effects (Fischer et al., 2022; Zhu et al., 2014).

The low levels of network specialisation (Agricultural mean H2’ = 0.397, Village/Urban mean H2’ = 0.390, Near‐Natural mean H2’ = 0.398) may reflect plasticity in host choice and the wide global distribution of suitable hosts. Host usage is characterised by a high degree of plasticity in biting insects and may be strongly influenced by host densities (Takken & Verhulst, 2013). For example, biting Diptera that are commonly described as anthropophilic such as *Anopheles gambiae* still interact with a range of domestic and non‐domestic hosts (Bellekom et al., 2021).

The low interaction evenness observed within Agricultural habitats, in comparison to other habitat types, indicates that interactions are dominated by relatively few species pairs, with a long tail of infrequently observed interactions. In Agricultural habitats, interactions involving domestic animals and humans dominated the networks, accounting for 81% of interactions, with cattle (51%) the most frequent hosts. This may reflect the high biomass of domestic animals (Lassen et al., 2012) in these habitats, and potentially the success and dominance of anthropophilic and livestock‐adapted biting Diptera species. Approximately 70% of biting Diptera species in the Agricultural networks fed predominantly (>50% of interactions) on humans and domestic animals, whilst the remaining Diptera fed on either a wider range of mammals and birds or had too few recorded blood meals to assess their diets with confidence (Bellekom et al., 2021). The minimal difference observed between our Agricultural model with targeted removal of domestic hosts and the null model with random removal may be explained by the high number of domestic hosts compared with non‐domestic hosts; random removal of hosts inevitably results in the removal of domestic hosts.

We found little difference in interaction evenness between Village/Urban and Near‐natural environments, although sampling was less complete within Near‐natural habitats. Despite an expected high human host availability, the Village/Urban networks were not dominated by interactions between humans and biting Diptera to the same extent as Agricultural habitats were by biting Diptera–cattle interactions. In Village/Urban habitats, 26% of interactions were with humans, and we identified interactions with a wide range of other taxa, predominantly birds and domesticated animals. Therefore, high interaction evenness values within Village/Urban sites may result from higher than expected generalism of biting Diptera (Hassell et al., 2017) and the absence of dominating species pairs (pairs with high abundance and interaction fidelity). In contrast, network interaction structure in Agricultural habitats is strongly influenced by the super‐abundance of a single suitable host species (cattle). Interaction evenness may therefore largely reflect host species evenness; independent data on host abundances would be required to test this.

For many taxa, specialist species are often more highly represented within pristine habitats, and are more susceptible to anthropogenic landscape modification and homogenisation than generalist species (Devictor, Julliard, & Jiguet, 2008; Devictor, Julliard, Clavel, et al., 2008; Sverdrup‐Thygeson et al., 2017). Despite this, we did not find differences in network specialism across levels of anthropogenic landscape modification, perhaps because the same genera and often the same species of biting Diptera were documented across habitat types, leading to similar feeding patterns.

Ecological networks are often asymmetric, composed of few strong interactions and a greater number of weak interactions (Poulin, 2010). Therefore, nodes likely affect each other with differing amounts of reciprocation, and the strength of an interaction is often determined by the distribution of abundance of the component species (Vázquez et al., 2007). Consequently, pairs of abundant species may exhibit more symmetric, and reciprocally strong, effects on the network than pairs of rare species (Dormann et al., 2017). Because independent abundance data were lacking for nodes within the networks, we considered all interactions to be inherently equivalent, with a normalised interaction strength of 1. These data limitations makes it impossible to identify the relative dynamic importance of different nodes. This is a common limitation in ecological network analyses of other interaction types, such as pollination (Novella‐Fernandez et al., 2019) and herbivory (Neff et al., 2021), in which the influence of each pollination visit or herbivory damage by different species is considered of equivalent impact on a plant. Network metrics can be sensitive to network size and species richness, leading to a risk that trends in cross‐network analyses reflect sampling differences, rather than genuine ecological patterns (Dormann et al., 2009). Heterogeneous data extracted from the literature are particularly susceptible to such biases as a result of variations in methods, sampling intensity and network dimensions (Prendergast & Ollerton, 2022; Xing & Fayle, 2021). Interaction evenness and network specialisation are relatively robust to network size differences (Blüthgen et al., 2006) and we included network size and species richness as explanatory variables in statistical models to control for this potential source of bias.

Agriculturally driven anthropogenic habitat modification, through its effects on biting Diptera–host interaction evenness and network specialism, could result in increased zoonotic disease transmission potential (McDaniel et al., 2014). There was a very high number of biting Diptera–bovine interactions in our Agricultural networks, involving a wide range of Diptera species from multiple families, many of which are vector‐competent. This may be of particular concern, since bovine‐related diseases such as Rift Valley fever, Animal African Trypanosomosis (nagana) and Bluetongue disease have high morbidity and mortality rates (Lopes et al., 2020; Rushton & Lyons, 2015; Vreysen et al., 2013). The growing global demand for agriculture products will result in continued anthropogenic habitat modification, which will provide increasing opportunities for pairwise interactions between unfamiliar species, zoonotic transmission and the emergence of novel zoonotic disease (Carlson et al., 2022). Surveillance of biting Diptera–host networks, particularly at the interface of humans, wildlife and domestic animals, could help identify pathways of zoonotic disease transmission and help predict and mitigate future spill‐over events. Surveillance may be conducted through the routine sampling of the Diptera community using a combination of trapping methods, such as malaise traps, USA Center for Disease Control (CDC) miniature light traps and Modified CDC Backpack Aspirators, Biogents' Sentinel (BGS) as well as trapping locations that limit accidental overrepresentation of a species in order to minimise sources of bias (Bellekom et al., 2021; Grubaugh et al., 2015; Gyawali et al., 2019; Rivera et al., 2021).

## AUTHOR CONTRIBUTIONS

**Ben Bellekom:** Conceptualization; data curation; formal analysis; investigation; methodology; project administration; validation; visualization; writing – original draft; writing – review and editing. **Owen T. Lewis:** Conceptualization; funding acquisition; supervision; writing – review and editing. **Talya D. Hackett:** Conceptualization; funding acquisition; supervision; writing – review and editing.

## CONFLICT OF INTEREST STATEMENT

The authors declare no conflicts of interest.

## Supporting information


**Figure S1.** Smoothed accumulation and extrapolation curves to assess sampling completeness. Total numbers of hosts (triangle), biting Diptera (circle), and interactions recorded in the whole dataset (square), by habitat type: Agricultural (blue), Near‐natural (red), and Village/Urban (orange), as a function of sampling effort (the number of blood meals screened).

**Figure S2.** Null model interaction evenness for each component network, with empirical IE values (red squares), by habitat type: Agricultural (a), Village/Urban (b), and Near‐natural (c). Each grey box displays the interquartile range, and the solid line represents the median values for interaction evenness. Whiskers display the maximum and minimum interaction evenness for each network.

## Data Availability

The data that support the findings of this study are available in Latitudinal‐and‐anthropogenic‐effects‐on‐the‐structuring‐of‐networks at https://github.com/Ben-Bellekom/Latitudinal-and-anthropogenic-effects-on-the-structuring-of-networks.
