# Supplementary material for: Latitudinal and anthropogenic effects on the structuring of networks linking blood‐feeding flies and their vertebrate hosts
Source: Med Vet Entomol. 2023 Jun 1;37(4):675–82. doi: 10.1111/mve.12671 (PMC10946476; doi:10.1111/mve.12671)
Supplement: Supplementary file 1 — Figure S1. Smoothed accumulation and extrapolation curves to assess sampling completeness. Total numbers of hosts (triangle), biting Diptera (circle), and interactions recorded in the whole dataset (square), by habitat type: Agricultural (blue), Near‐natural (red), and Village/Urban (orange), as a function of sampling effort (the number of blood meals screened). [file MVE-37-675-s001.docx]

Figure S1. Smoothed accumulation and extrapolation curves to assess sampling completeness. Total numbers of hosts (triangle), biting Diptera (circle), and interactions recorded in the whole dataset (square), by habitat type: Agricultural (blue), Near-natural (red), and Village/Urban (orange), as a function of sampling effort (the number of blood meals screened).
