# Supplementary material for: Latitudinal and anthropogenic effects on the structuring of networks linking blood‐feeding flies and their vertebrate hosts
Source: Med Vet Entomol. 2023 Jun 1;37(4):675–82. doi: 10.1111/mve.12671 (PMC10946476; doi:10.1111/mve.12671)
Supplement: Supplementary file 2 — Figure S2. Null model interaction evenness for each component network, with empirical IE values (red squares), by habitat type: Agricultural (a), Village/Urban (b), and Near‐natural (c). Each grey box displays the interquartile range, and the solid line represents the median values for interaction evenness. Whiskers display the maximum and minimum interaction evenness for each network. [file MVE-37-675-s002.docx]

**
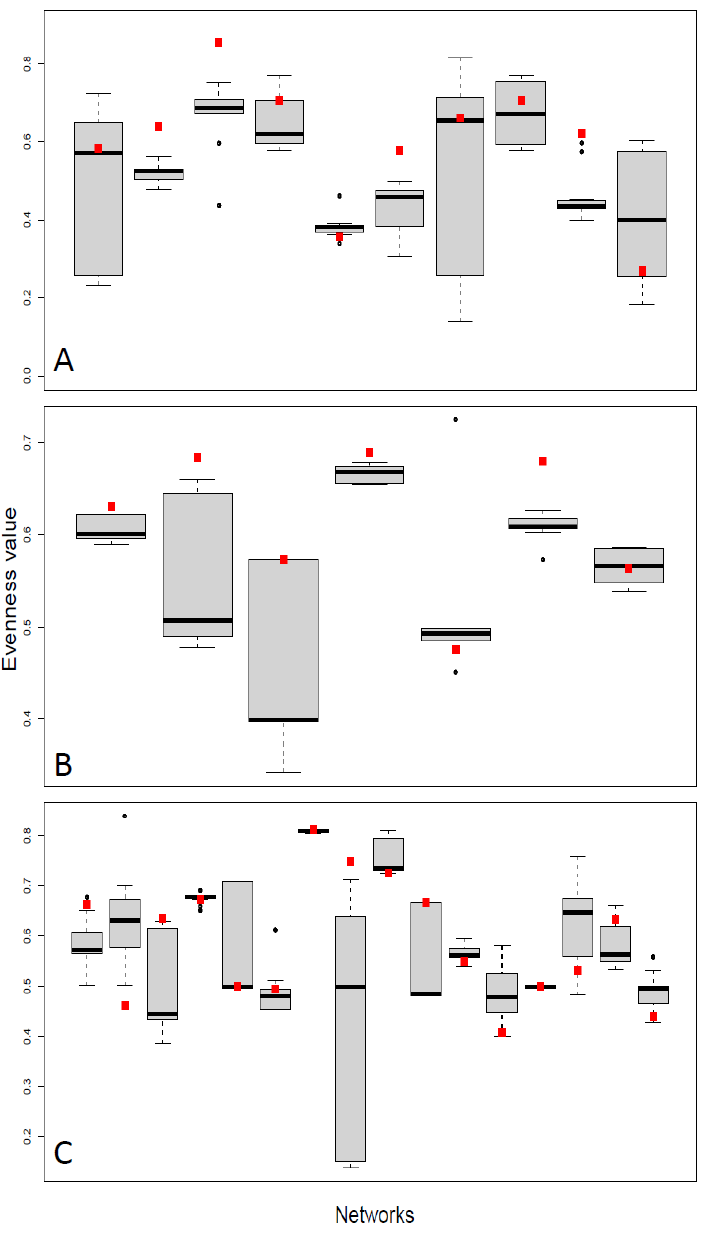
**

Figure S2. Null model interaction evenness for each component network, with empirical IE values (red squares), by habitat type: Agricultural (A), Village/Urban (B), and Near-natural (C). Each grey box displays the interquartile range, and the solid line represents the median values for interaction evenness. Whiskers display the maximum and minimum interaction evenness for each network.
